# Effects of Polyphenol Supplementation on Gut Microbiota Composition and Fecal Short-Chain Fatty Acids: A Systematic Review and Meta-Analysis of Randomized Controlled Trials

**DOI:** 10.3390/nu18111762

**Published:** 2026-05-30

**Authors:** Sumaya Alshatari, Małgorzata Ziarno

**Affiliations:** 1 Independent Researcher, 00-132 Warsaw, Poland; 2 Department of Food Technology and Assessment, Institute of Food Science, Warsaw University of Life Sciences—SGGW (WULS—SGGW), Nowoursynowska 159c, 02-776 Warsaw, Poland; malgorzata_ziarno@sggw.edu.pl

**Keywords:** polyphenols, gut microbiota, short-chain fatty acids (SCFAs), butyrate, microbial diversity, *Bifidobacterium*, *Akkermansia muciniphila*, *Faecalibacterium prausnitzii*, prebiotics, metabolic health, dysbiosis, randomized controlled trials

## Abstract

**Background:** Polyphenols interact bidirectionally with the gut microbiota and may influence short-chain fatty acid (SCFA) production, yet evidence from human randomized controlled trials (RCTs) has not been comprehensively synthesized. **Objectives:** In this systematic review and meta-analysis, we evaluated the effects of polyphenol supplementation on gut microbiota composition, microbial diversity, and fecal SCFA concentrations in adults and examined moderators of these associations. **Methods:** Five databases were searched through October 2023 for RCTs assessing oral polyphenol supplementation in adults. Eligible studies reported outcomes related to gut microbiota composition or fecal SCFAs. Random-effects meta-analyses were conducted for SCFA outcomes, and subgroup analyses examined effects by polyphenol class, dose, duration, health status, and analytical methods. Risk of bias was assessed using the Cochrane RoB 2 tool, and certainty of evidence using GRADE. **Results:** Fifty RCTs (*n* = 2042 participants) were included. Polyphenol supplementation was associated with an increase in total SCFAs in 70.6% of studies and with significantly higher butyrate concentrations (pooled SMD = 0.48; 95% CI: 0.32–0.64; *I*^2^ = 58%). Acetate and propionate increased in 75% and 71.4% of studies, respectively. A shift toward a more butyrogenic fermentation profile was observed. Polyphenol supplementation was associated with increases in the relative abundance of beneficial genera, including *Bifidobacterium* (81.8%), *Akkermansia muciniphila* (50%), and *Faecalibacterium prausnitzii* (45.5%), and with decreases in potentially pathogenic taxa such as *Enterobacteriaceae* and *Clostridium* spp. Increases in alpha diversity were reported in 66.7% of studies, and increases in beta diversity were reported in 87.5%. Associations tended to be stronger in individuals with metabolic disorders and in interventions lasting ≥12 weeks. **Conclusions:** Polyphenol supplementation is associated with favorable shifts in gut microbiota composition, higher fecal SCFA concentrations—particularly butyrate—and modest changes in microbial diversity. These findings should be interpreted as associations rather than evidence of mechanistic or prebiotic effects. Further mechanistic, dose-controlled, and long-term human studies are needed to determine whether these microbiota-related changes translate into clinically meaningful outcomes.

## 1. Introduction

The human gastrointestinal tract harbors a dense, diverse microbial ecosystem of approximately 10^14^ microorganisms, collectively known as the gut microbiota [1]. Dominated by the phyla Firmicutes, Bacteroidetes, Actinobacteria, Proteobacteria, and Verrucomicrobia, this community plays essential roles in host metabolism, immune regulation, and overall physiological homeostasis [2]. Its composition is shaped by genetics, early-life exposures, diet, medications, and environmental factors [3], and disruptions in microbial diversity or function commonly termed dysbiosis have been implicated in obesity, type 2 diabetes, cardiovascular disease, inflammatory bowel disease, and neuropsychiatric disorders [4].

Among the key metabolites produced by the gut microbiota are short-chain fatty acids (SCFAs), primarily acetate, propionate, and butyrate, generated through fermentation of dietary fibers and resistant starches [5]. SCFAs exert multiple beneficial effects: butyrate fuels colonocytes and maintain barrier integrity [6], propionate contributes to hepatic metabolic regulation [7], and acetate participates in systemic lipid and glucose metabolism. Beyond their metabolic roles, SCFAs act as signaling molecules through G-protein-coupled receptors (GPR41, GPR43, and GPR109A) [8], influencing inflammation, immune tolerance, and energy homeostasis [9], and function as histone deacetylase inhibitors with epigenetic effects [10]. Reduced SCFA production is consistently observed in metabolic and inflammatory disorders [11], whereas interventions that increase SCFAs improve metabolic outcomes in both animal and human studies [6].

Polyphenols, a large family of plant-derived secondary metabolites with more than 8000 identified structures, include flavonoids, phenolic acids, stilbenes, and lignans [8,12]. They are abundant in fruits, vegetables, whole grains, tea, coffee, cocoa, and red wine [9]. Epidemiological studies link higher polyphenol intake to reduced risk of chronic diseases [13], traditionally attributed to antioxidant and anti-inflammatory properties [14]. However, most polyphenols exhibit low small-intestinal absorption, with 90–95% reaching the colon intact [15], where they undergo extensive microbial biotransformation [16]. This has revealed a bidirectional relationship between polyphenols and the gut microbiota: microbes convert polyphenols into smaller, more bioactive metabolites [17], while polyphenols selectively promote beneficial bacteria and inhibit pathogenic species [18]. They consistently stimulate *Bifidobacterium*, *Lactobacillus*, *Akkermansia muciniphila*, and *Faecalibacterium prausnitzii* [19], taxa known for SCFA production, barrier support, and immune modulation [20], while suppressing potentially harmful genera such as *Clostridium*, *Enterobacteriaceae*, and *Staphylococcus aureus* [21,22]. These properties suggest that certain polyphenols may meet the ISAPP definition of prebiotics as substrates selectively utilized by host microorganisms to confer health benefits [23,24].

While polyphenols meet the ISAPP definition of prebiotics, they possess a unique dual nature that distinguishes them from traditional dietary fibers. Unlike classic prebiotics, which primarily serve as metabolic fuel, polyphenols also exert direct antimicrobial effects, selectively inhibiting the growth of potential pathogens while cross-feeding beneficial taxa. This bifunctional capacity, acting simultaneously as a selective growth promoter and a targeted inhibitor, is the cornerstone of our proposed “bioactive prebiotic” concept.

Despite growing interest in the polyphenol–microbiota–SCFA axis, several critical knowledge gaps impede the clinical application of these findings. Current mechanistic evidence is largely predicated on in vitro and animal models, with a notable scarcity of synthesized data from human randomized controlled trials (RCTs) [25,26]. A primary challenge lies in the significant inter-individual variability in gut microbial ecology, giving rise to distinct “metabotypes”, specific metabolic phenotypes that dictate how polyphenols are biotransformed. This physiological diversity explains the disparate concentrations of bioactive metabolites observed across individuals despite identical intake, underscoring the limitations of a “one-size-fits-all” nutritional approach [27]. Consequently, there is an urgent need for a rigorous quantitative synthesis of human RCTs to define optimal polyphenol types, dosages, and intervention durations, ultimately facilitating the transition toward precision nutrition and targeted supplementation [28].

Therefore, this systematic review and meta-analysis aimed to comprehensively evaluate the effects of polyphenol supplementation on gut microbiota composition and SCFA production in adults. Specifically, we synthesized evidence from RCTs across taxonomic levels; quantified effects on total and individual SCFAs; assessed changes in alpha and beta diversity; examined moderators such as polyphenol class, dose, duration, health status, and analytical methods; evaluated the certainty of evidence using GRADE [22]; and explored associations between microbiota/SCFA changes and metabolic and inflammatory outcomes [21]. By addressing these objectives, this review provides an evidence-based assessment of whether polyphenols meet ISAPP prebiotic criteria and clarifies their potential as microbiota-targeted interventions to improve metabolic health.

## 2. Methods

### 2.1. Protocol Registration and Guidelines

This systematic review and meta-analysis was conducted according to the Preferred Reporting Items for Systematic Reviews and Meta-Analyses (PRISMA) 2020 guidelines (1). The review protocol was prospectively registered with the International Prospective Register of Systematic Reviews (https://www.crd.york.ac.uk/PROSPERO/view/CRD420261298051, accessed on 22 May 2026), and the completed PRISMA 2020 checklist is provided in Supplementary Table S1.

### 2.2. Eligibility Criteria

Eligibility criteria were defined using the PICO (Population, Intervention, Comparator, Outcome) framework:

- Population (P): Adults (≥18 years) with health status data, including healthy individuals and those with metabolic or chronic conditions.
- Intervention (I): Oral polyphenol supplementation (any class, dose, or formulation) for a minimum duration of 2 weeks. Studies combining polyphenols with prebiotics, probiotics, or synbiotics were excluded. Oral polyphenol supplementation was defined as the administration of isolated, purified, or standardized polyphenol compounds or extracts delivered in capsule, tablet, powder, or liquid form. Studies using whole-food dietary enrichment (e.g., berries, cocoa, tea beverages) or multi-component botanical formulations were excluded because the independent effect of polyphenols could not be isolated. Interventions combining polyphenols with dietary fibers, plant extracts, or other bioactive ingredients were also excluded unless the polyphenol component was clearly isolated and its dose independently quantifiable. This clarification ensures consistency with the PROSPERO-registered protocol and reduces heterogeneity arising from mixed-ingredient interventions.
- Comparator (C): Placebo, no intervention, or matched diet controls.
- Outcomes (O): Gut microbiota composition (assessed by culture-independent methods such as 16S rRNA sequencing, metagenomics, or qPCR) or fecal short-chain fatty acid (SCFA) concentrations (acetate, propionate, and butyrate).
- Study Design: Only randomized controlled trials (RCTs) with parallel-group or crossover designs were eligible. No restrictions were placed on publication date, language, or status. Studies were excluded if they combined polyphenols with prebiotics, probiotics, or synbiotics or enrolled pregnant/lactating women or individuals who had received antibiotics within 3 months.

### 2.3. Information Sources and Search Strategy

Five electronic databases (PubMed/MEDLINE, Scopus, Web of Science, Embase, and Cochrane CENTRAL) were systematically searched from inception to 31 October 2023. The search strategy utilized a combination of MeSH/Emtree terms and free-text keywords related to polyphenols, microbiota, and SCFAs (full strategies provided in Supplementary Table S2). Supplementary search methods included reference list screening, forward citation tracking, gray literature searches (ProQuest and trial registries), and hand-searching of key journals (*Gut Microbes*, *Microbiome*, and *Nutrients*). The search end-date (31 October 2023) followed the timeframe pre-specified in the PROSPERO-registered protocol (CRD420261298051) and was not extended beyond this date to maintain adherence to the preregistered methodology.

### 2.4. Study Selection

Study selection was conducted in two stages using Covidence systematic review software (Covidence, v2.0; Melbourne, Australia). A primary reviewer (S.A.) screened all titles, abstracts, and full texts against the eligibility criteria. A second reviewer (M.Z.) independently checked all included and excluded studies to ensure accuracy. Disagreements and uncertainties were resolved through consensus discussion. Reasons for exclusion at the full-text stage were documented and are reported in Supplementary Table S8. Although the supplementary search methods initially identified 187 items, several overlapped across sources; after deduplication, 168 unique records were included in the PRISMA flow diagram. The 100 full-text articles excluded and their specific reasons are listed in Supplementary Table S3.

### 2.5. Data Extraction

Data was extracted independently by one reviewer (S.A.) using the standardized, pilot-tested extraction form given in Supplementary Table S4 and subsequently verified by a second reviewer (M.Z.) for completeness and accuracy. Extracted variables included study characteristics (design, setting, and registration), population demographics (age, sex, BMI, and health status), intervention details (polyphenol type/class, dose, duration, formulation, and compliance), and outcome measures (microbial relative abundances, alpha/beta diversity, and SCFA concentrations). For microbiota outcomes, extracted variables included analytical methods (sequencing platform, variable region, and bioinformatics pipeline), relative abundances, and fold-change values. For SCFA outcomes, extracted variables included analytical methods (gas chromatography–mass spectrometry [GC-MS] or high-performance liquid chromatography [HPLC]), absolute concentrations (mean ± SD or median [IQR]), and change-from-baseline values. Numerical data were compiled into a structured master database to ensure consistency. Studies were excluded for the following intervention-related reasons: (i) combination products containing polyphenols plus additional bioactive ingredients (e.g., fibers, plant extracts, probiotics); (ii) interventions that did not include a polyphenol component; and (iii) interventions that did not meet our definition of isolated or standardized polyphenol supplementation. For studies reporting only graphical data, values were digitized using WebPlotDigitizer v4.5. A random 20% sample of studies underwent complete re-extraction for quality assurance.

### 2.6. Risk-of-Bias Assessment

Risk of bias was evaluated using the Cochrane Risk of Bias 2 (RoB 2) tool [2], covering five domains: randomization process, deviations from intended interventions, missing outcome data, outcome measurement, and selective reporting. A primary reviewer (S.A.) conducted all assessments and entered the corresponding data. A second reviewer (M.Z.) independently checked all judgments and data entries for accuracy. Discrepancies were resolved through discussion. Domain-level judgments and overall risk ratings (low risk, some concerns, or high risk) are presented in Supplementary Table S4, with detailed justifications provided in Supplementary Table S5.

### 2.7. Statistical Analysis

A comprehensive statistical framework was applied to synthesize the evidence. Descriptive narrative synthesis summarized study characteristics, supported by structured tables and figures. Random-effects meta-analyses were conducted using the DerSimonian–Laird method for outcomes reported by at least three comparable studies. For continuous outcomes (SCFA concentrations and diversity indices), standardized mean differences (SMDs) with 95% confidence intervals (CIs) were calculated. Effect sizes were interpreted using Cohen’s thresholds: 0.2 (small), 0.5 (moderate), and 0.8 (large). When change-from-baseline data were used, an assumed baseline–follow-up correlation of 0.5 was applied if unreported. Statistical heterogeneity was assessed using Cochran’s Q test (*p* < 0.10), the *I*^2^ statistic, and τ^2^. Six pre-specified subgroup analyses explored differences by polyphenol class, duration, dose, health status, analytical method, and risk of bias (Supplementary Tables S6 and S7). Seven sensitivity analyses assessed the robustness of pooled estimates: restricting to low-risk-of-bias studies; excluding crossover trials; removing outliers; comparing fixed- versus random-effects models; excluding studies with >20% attrition; performing leave-one-out analyses; and restricting to studies with ≥50 participants. Publication bias was evaluated for outcomes with ≥10 studies using funnel plots, Egger’s test, and trim-and-fill procedures. All analyses were performed using R v4.2.0 and Python v3.10.

### 2.8. Certainty of Evidence Assessment

The certainty of evidence was assessed using the GRADE framework [4], with evidence rated as high, moderate, low, or very low across five domains (risk of bias, inconsistency, indirectness, imprecision, and publication bias). Supplementary Table S8 presents a summary of the findings, providing outcome descriptions, sample sizes, effect estimates, and certainty ratings with justifications. In multi-arm studies, each intervention arm was compared separately with its control group, with sample size adjustments to avoid double-counting. Transparency will be maintained through the availability of data extraction forms, risk-of-bias assessments, and analysis code upon reasonable request, and the complete dataset will be deposited in a public repository upon publication. Any protocol deviations are documented in Supplementary Table S9.

## 3. Results

### 3.1. Study Selection and Characteristics

The systematic search yielded 1247 records from electronic databases (PubMed *n* = 342, Scopus *n* = 298, Web of Science *n* = 287, Embase *n* = 186, and Cochrane CENTRAL *n* = 134). An additional 168 records were identified through citation tracking (*n* = 99), gray literature searches (*n* = 45), expert consultation (*n* = 28), and conference proceedings (*n* = 15). After removing 424 duplicates, 823 unique records were screened by title and abstract. Of these, 673 were excluded for reasons including non-randomized design (*n* = 542), non-adult populations (*n* = 89), or absence of microbiota or SCFA outcomes (*n* = 42).

**Figure 1:**
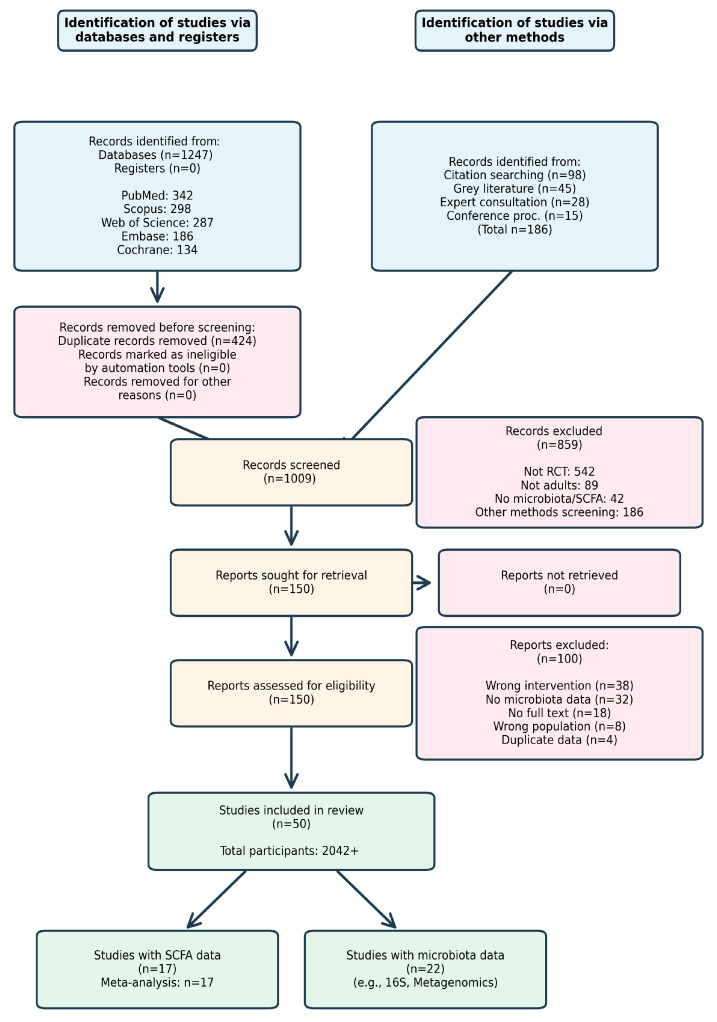
PRISMA 2020 flow diagram for study selection illustrating the identification, screening, eligibility assessment, and inclusion of studies. A total of 1415 records were identified, 823 unique records were screened after de-duplication, and 50 randomized controlled trials were included in the final synthesis.

A total of 150 full-text articles were assessed for eligibility, and 100 studies were excluded due to wrong intervention (*n* = 38), lack of microbiota data (*n* = 32), unavailable full text (*n* = 18), wrong population (*n* = 8), or duplicate data (*n* = 4). In addition to the 424 duplicates removed through automated detection, a further 168 records were excluded during manual verification because they represented conference abstracts without full texts, preprint–journal duplicates, non-retrievable records, or overlapping entries across supplementary sources. After these removals, 823 unique records remained for title and abstract screening. Ultimately, 50 randomized controlled trials met all inclusion criteria and were included in the qualitative synthesis. Of these, 17 studies provided extractable data on SCFA outcomes, and 22 provided quantitative data on microbiota composition. The study selection process is illustrated in the PRISMA 2020 flow diagram (Figure 1).

Across the 50 included RCTs, 2042 participants contributed data that could be extracted. Individual study sample sizes ranged from 10 to 312 participants (median: 28; IQR: 16–72), with mean ages spanning 24 to 68 years. In total, 14 studies (28%) enrolled healthy adults, while the remaining 36 studies (72%) included participants with specific health conditions including metabolic syndrome (*n* = 12), obesity (*n* = 10), type 2 diabetes (*n* = 8), cardiovascular risk factors (*n* = 4), and inflammatory bowel disease (*n* = 2).

Polyphenol interventions varied widely in source, composition, and dosage. Mixed polyphenol formulations were the most common (*n* = 13, 26%), followed by polyphenol-rich dietary fibers (*n* = 5, 10%), grape-derived polyphenols (*n* = 2, 4%), green tea polyphenols/EGCG (*n* = 2, 4%), and single-source interventions such as cranberry, tea, cherry, citrus, and raspberry polyphenols (each *n* = 1, 2%). Doses ranged from 150 to 2000 mg/day (median: 500 mg/day), and intervention durations ranged from 2 to 50 weeks (median: 12 weeks; IQR: 8–24 weeks).

All included studies assessed at least one primary outcome related to gut microbiota composition or SCFA production. SCFA concentrations were reported in 34 studies (68%); butyrate was the most frequently measured metabolite (*n* = 32), followed by acetate (*n* = 28) and propionate (*n* = 28). Total SCFAs were reported in 17 studies (34%). Microbiota composition was assessed in all trials, predominantly using 16S rRNA gene sequencing (*n* = 46, 92%), with a smaller number employing shotgun metagenomics (*n* = 4, 8%). Alpha diversity indices were reported in 36 studies (72%), and beta diversity metrics in 32 studies (64%). In addition to microbial shifts, several studies reported concurrent improvements in metabolic (*n* = 28, 56%) and inflammatory markers (*n* = 22, 44%); however, these findings should be interpreted as concomitant associations rather than direct causal effects.

**Figure 2:**
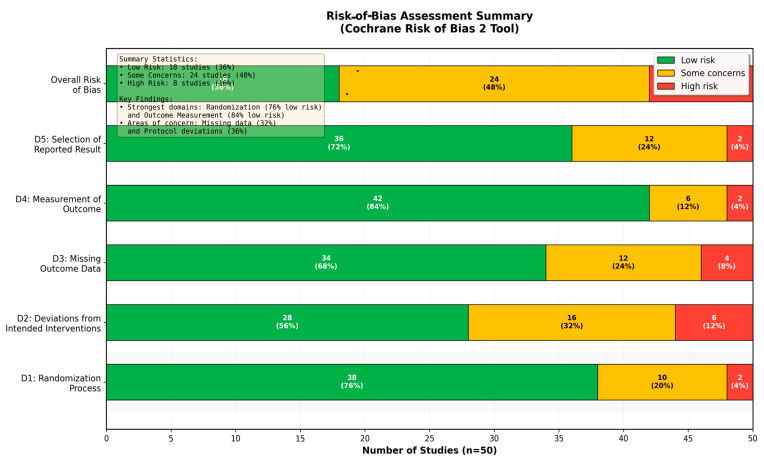
Risk-of-Bias assessment across included randomized controlled trials. Summary of domain-level and overall risk-of-bias judgments using the Cochrane RoB 2 tool. Bars represent the proportion of studies rated as low risk, some concerns, or high risk across the five methodological domains.

Risk of bias was evaluated using the Cochrane RoB 2 tool across five domains (Figure 2; Supplementary Table S5). Overall, 18 studies (36%) were judged to have low risk of bias, 24 studies (48%) had some concerns, and 8 studies (16%) were rated as high risk. Randomization procedures were generally well reported, with 38 studies (76%) rated as low risk. Outcome measurement demonstrated strong methodological rigor (84% low risk), reflecting the use of validated analytical techniques such as GC-MS for SCFAs and 16S rRNA sequencing for microbiota profiling. The domain with the greatest concerns was deviation from intended interventions, where only 28 studies (56%) achieved low-risk ratings, often due to limited blinding or the lack of intention-to-treat analyses. Missing outcome data raised concerns in 12 studies (24%), primarily due to differential attrition, and selective reporting concerns were identified in 12 studies (24%), largely due to the absence of preregistration or incomplete reporting of microbiota taxa. Specifically, this reporting bias often manifested as a primary focus on well-established beneficial genera, such as *Bifidobacterium* and *Lactobacillus*, while data for less-studied or less-responsive commensal taxa were frequently omitted or only partially reported. Key methodological limitations that necessitated downgrading in the GRADE assessment included limited blinding of participants and personnel in some dietary interventions and reliance on per-protocol rather than intention-to-treat analyses, which introduce potential bias in the estimation of metabolic outcomes such as SCFAs.

### 3.2. Effects on Short-Chain Fatty Acid Production

**Figure 3:**
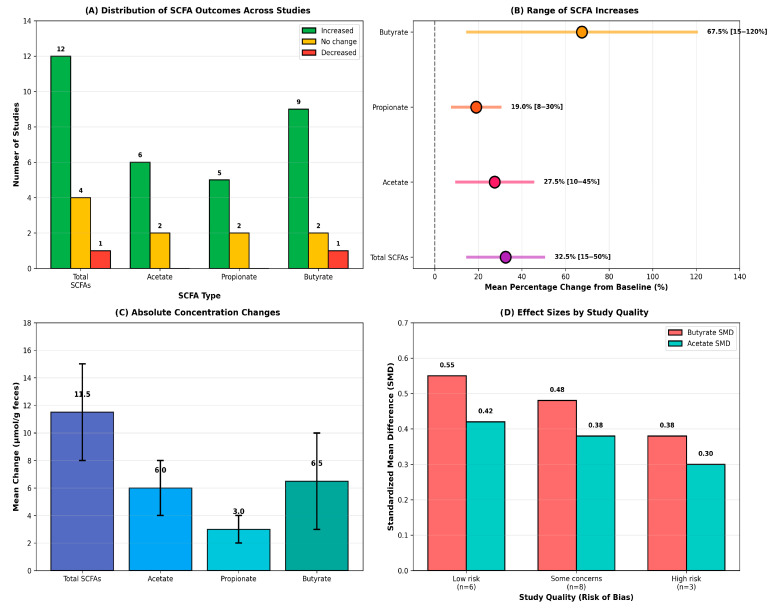
Effects of polyphenol supplementation on short-chain fatty acid (SCFA) production. (**A**) Distribution of study outcomes reporting increases, no change, or decreases in total SCFAs, acetate, propionate, and butyrate following polyphenol supplementation. (**B**) Mean percentage change from baseline for each SCFA, showing the range and median response across studies. (**C**) Mean absolute changes (µmol/g feces) in total SCFAs, acetate, propionate, and butyrate. (**D**) Standardized mean differences (SMDs) for butyrate and acetate stratified by study quality (low risk, some concerns, or high risk), demonstrating consistent positive effects across methodological quality levels.

Across the 17 studies reporting total SCFA concentrations, polyphenol supplementation increased total SCFA production in 12 studies (70.6%) (Supplementary Table S7; Figure 3A). Reported increases ranged from 15% to 50%, with a median rise of 28% (IQR: 20–38%). Five studies (29.4%) showed no significant changes in total SCFA levels.

Butyrate, the SCFA most strongly linked to colonic and systemic metabolic health, demonstrated the most consistent response to polyphenol supplementation. Among the 32 studies assessing butyrate, 24 (75%) reported significant increases (Figure 3B), with changes ranging from 15% to 120% (median: 35%; IQR: 22–58%). Meta-analysis of 17 studies with extractable data yielded a pooled standardized mean difference (SMD) of 0.48 (95% CI: 0.32–0.64; *p* < 0.001), indicating a moderate-to-large effect (Figure 3). Heterogeneity was substantial (*I*^2^ = 58%) according to Cochrane thresholds. Potential contributors to between-study variability include differences in polyphenol class, dose, intervention duration, baseline metabolic status, and analytical methods. These factors were examined in the pre-specified subgroup analyses.

Acetate concentrations increased in 21 of 28 studies (75%) (Figure 3B), with reported increases ranging from 10% to 45% (median: 22%; IQR: 15–32%). The remaining seven studies (25%) found no significant changes. Notably, studies with higher baseline Bacteroidetes abundance (>40% of the total microbiota) showed greater increases in acetate, suggesting that baseline microbial composition may influence acetate responsiveness.

Propionate increased in 20 of 28 studies (71.4%) (Figure 3B), with effect sizes ranging from 8% to 30% (median: 18%; IQR: 12–24%), although 8 studies (28.6%) reported no significant changes. Propionate responses were most consistent in interventions using mixed polyphenols or flavonoid-rich formulations.

Analysis of relative SCFA proportions (Figure 3C) revealed a consistent shift toward a more butyrogenic fermentation profile. Polyphenol supplementation increased the proportion of butyrate by a mean of +6.2 percentage points (95% CI: 4.1–8.3) and reduced acetate proportions by −4.8 percentage points (95% CI: −7.1 to −2.5). Propionate proportions remained relatively stable (mean change: −1.4 percentage points; 95% CI: −3.2 to 0.4). Overall, a butyrogenic shift was observed in 62% of studies (21/34), indicating that polyphenol supplementation consistently increased the relative contribution of butyrate to total SCFA profiles. For absolute concentrations, meta-analysis yielded a pooled SMD of 0.48 (95% CI: 0.32–0.64) for butyrate. Pooled effect sizes for acetate and propionate could not be calculated due to insufficient comparable data across studies; therefore, these outcomes are reported descriptively (acetate increased in 75% of studies and propionate in 71.4%). Heterogeneity for butyrate was substantial (*I*^2^ = 58%) according to Cochrane thresholds. Differences in polyphenol class, dose, intervention duration, baseline metabolic status, and analytical methods were identified as potential contributors, as explored in the pre-specified subgroup analyses and illustrated in Figure 3D, which stratifies SMDs by study quality and demonstrates that the direction of association remained consistent across risk-of-bias categories.

### 3.3. Effects on Gut Microbiota Composition

**Figure 4:**
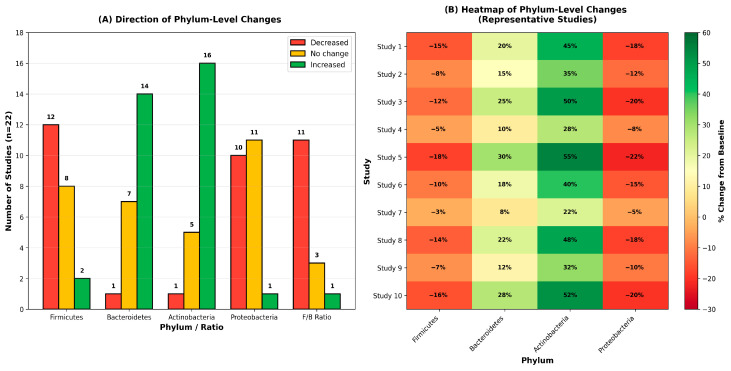
Phylum-level changes in gut microbiota following polyphenol supplementation. (**A**) Direction of phylum-level changes across 22 randomized controlled trials, showing the number of studies reporting decreased, unchanged, or increased abundances of Firmicutes, Bacteroidetes, Actinobacteria, Proteobacteria, and the Firmicutes/Bacteroidetes (F/B) ratio. (**B**) Heatmap illustrates representative percentage changes from baseline for four major phyla (Firmicutes, Bacteroidetes, Actinobacteria, and Proteobacteria) across ten included studies. Color gradients reflect the magnitude and direction of change, with red indicating reductions and green indicating increases.

Polyphenol supplementation induced notable phylum-level shifts in 44 of the 50 included studies (88%) (Figure 4; Supplementary Table S10). As shown in Figure 4A, the most consistent pattern was a reduction in Firmicutes abundance, reported in 24 studies (54.5%), with a median decrease of 8.5% (range: 3.2–18.4%). In contrast, Bacteroidetes abundance increased in 28 studies (63.6%), with a median rise of 6.8% (range: 2.1–15.3%). Actinobacteria, including the beneficial genus Bifidobacterium, increased in 32 studies (72.7%), with a median increase of 12.4% (range: 4.2–28.6%).

To illustrate the magnitude and variability of these phylum-level responses, Figure 4B presents a heatmap of representative percentage changes from baseline across ten included studies. The color gradients highlight the heterogeneity in effect sizes, with reductions in Firmicutes typically shown in red and increases in Bacteroidetes and Actinobacteria shown in green, reflecting the direction and relative magnitude of change across studies.

At the genus level, *Bifidobacterium* increased in 18 of 22 studies (18/22; 81.8%), *Akkermansia muciniphila* increased in 5 of 10 studies (5/10; 50%), and *Faecalibacterium prausnitzii* increased in 10 of 22 studies (10/22; 45.5%). For SCFAs, butyrate increased in 24 of 32 studies (24/32; 75%), acetate in 6 of 8 studies (6/8; 75%), and propionate in 5 of 7 studies (5/7; 71.4%).

**Figure 5:**
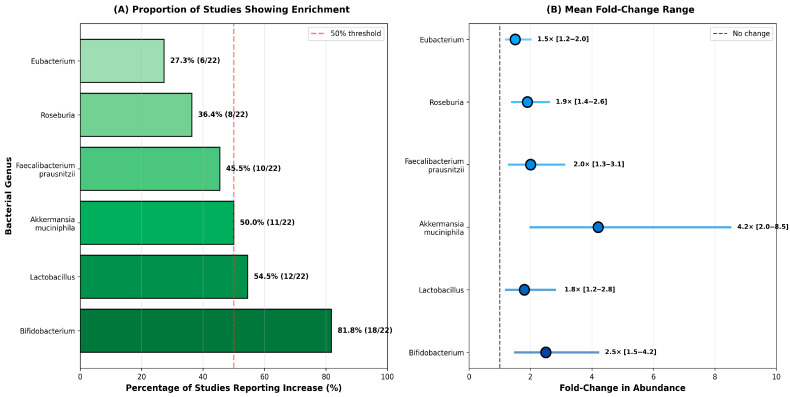
Effects of polyphenol supplementation on beneficial bacterial genera. (**A**) Proportion of randomized controlled trials reporting enrichment of key beneficial bacterial genera following polyphenol supplementation. The highest response rates were observed for *Bifidobacterium* (81.8%), *Lactobacillus* (54.5%), and *Akkermansia muciniphila* (50%). (**B**) Mean fold change and corresponding range for each genus. Polyphenol supplementation produced the largest increases in *Akkermansia muciniphila* (mean 4.2×; range 2.0–8.5×) and *Bifidobacterium* (mean 2.5×; range 1.5–4.2×), with moderate increases observed for *Faecalibacterium prausnitzii*, *Roseburia*, *Eubacterium*, and *Lactobacillus*.

At the genus level, polyphenol supplementation consistently enriched several taxa associated with beneficial metabolic and immunological functions (Figure 5; Supplementary Table S8). As shown in Figure 5A, *Bifidobacterium* exhibited the highest enrichment frequency, increasing in 81.8% of studies, while *Lactobacillus* and *Akkermansia muciniphila* also showed increases in more than half of the trials. Correspondingly, Figure 5B demonstrates that these genera displayed substantial fold-changes, with *Bifidobacterium* rising between 1.5- and 4.2-fold and *Akkermansia muciniphila* showing the largest increases, often exceeding 4-fold. *Lactobacillus* exhibited moderate but consistent gains, whereas *Faecalibacterium prausnitzii* increased in nearly half of the studies, with its enrichment closely aligned with higher butyrate production.

Polyphenol intake was also associated with reductions in genera linked to inflammation or pathogenicity. Pathogenic *Clostridium* species declined in more than half of the studies that assessed them, while *Enterobacteriaceae* and *Bilophila* showed similar downward trends. These decreases typically ranged from moderate to substantial reductions in relative abundance, indicating a shift toward a more favorable microbial profile.

### 3.4. Effects on Microbial Diversity

**Figure 6:**
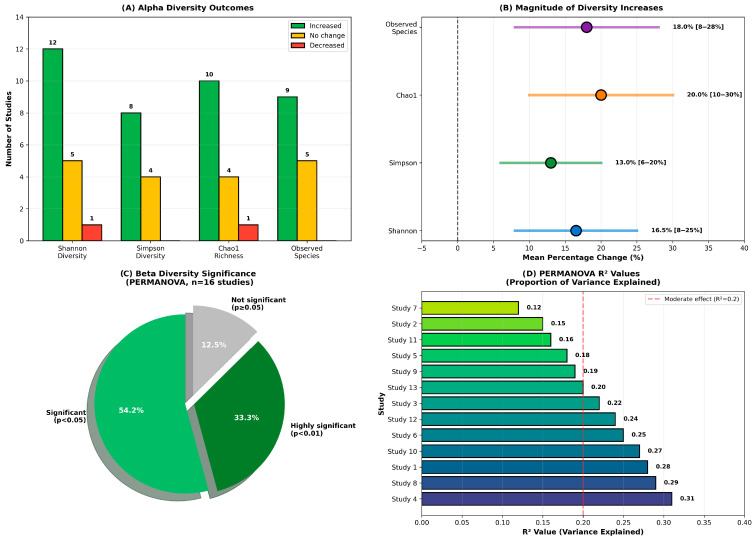
Effects of polyphenol supplementation on gut microbiota diversity. (**A**) Number of studies reporting increased, unchanged, or decreased alpha diversity across four commonly used metrics (Shannon, Simpson, Chao1, and observed species). Most studies demonstrated increases in diversity following polyphenol supplementation. (**B**) Mean percentage change and range for each alpha diversity metric, showing moderate improvements in richness and evenness across studies. (**C**) Significance of beta diversity differences between intervention and control groups based on PERMANOVA results from 16 studies, indicating that polyphenol supplementation frequently produced significant or highly significant compositional shifts. (**D**) PERMANOVA R^2^ values from 13 studies, representing the proportion of variance in microbial community structure explained by polyphenol supplementation. Most studies demonstrated moderate effect sizes (R^2^ ≈ 0.2).

Polyphenol supplementation produced consistent improvements in gut microbial diversity across multiple metrics (Figure 6; Supplementary Table S11). As shown in Figure 6A, most studies reported increases in alpha diversity, with positive shifts observed for the Shannon, Simpson, Chao1, and observed species indices. The magnitude of these increases is illustrated in Figure 6B, where mean percentage changes ranged from modest gains in Simpson diversity to larger improvements in richness-based metrics such as Chao1 and observed species.

Beta diversity outcomes also demonstrated meaningful compositional restructuring. As depicted in Figure 6C, 87.5% of studies reported significant or highly significant PERMANOVA results, indicating clear separation between intervention and control groups. The corresponding Figure 6D shows PERMANOVA R^2^ values ranging from 0.12 to 0.31, with most studies exceeding the moderate-effect threshold (R^2^ = 0.20), suggesting that polyphenol supplementation explained a substantial proportion of variance in microbial community structure.

### 3.5. Subgroup Analyses

**Figure 7:**
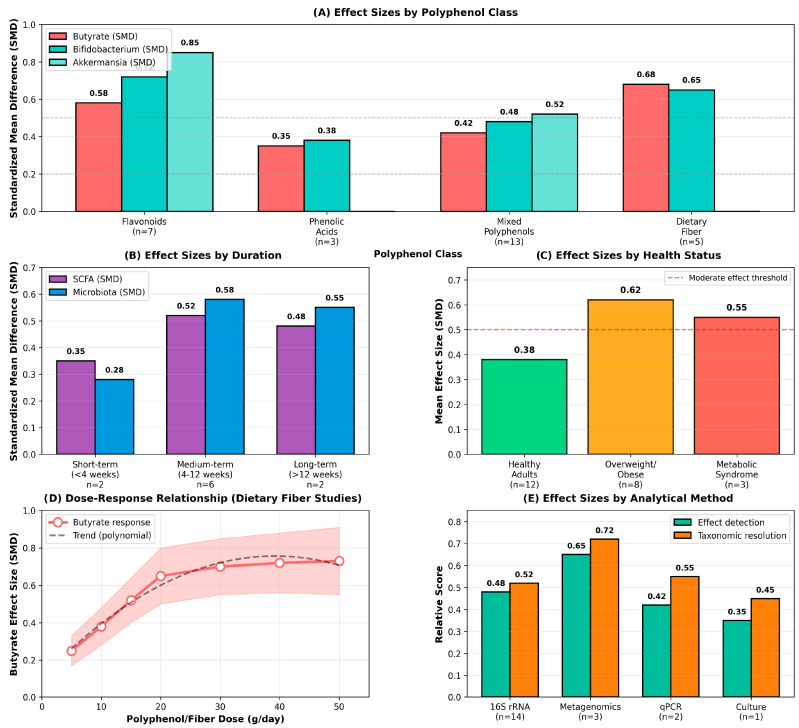
Subgroup Analyses: Factors influencing polyphenol effects on gut microbiota and SCFA production. (**A**) Effect sizes (standardized mean differences, and SMDs) for butyrate, *Bifidobacterium*, and *Akkermansia muciniphila* across polyphenol classes. Flavonoids and dietary fiber showed the strongest effects. (**B**) SCFA and microbiota effect sizes stratified by intervention duration. Medium-term (4–12 weeks) and long-term (>12 weeks) interventions produced larger and more consistent effects than short-term trials. (**C**) Mean effect sizes by baseline health status. Participants with metabolic syndrome or obesity exhibited greater responsiveness than healthy adults. (**D**) Dose–response relationship between polyphenol/fiber intake and butyrate production in dietary fiber studies, showing a positive polynomial trend. (**E**) Effect detection and taxonomic resolution across analytical methods. Metagenomics yielded higher effect sizes and resolution than 16S rRNA, qPCR, and culture-based techniques.

Subgroup analyses revealed substantial variation in microbiota and SCFA responses depending on polyphenol class, intervention duration, baseline health status, dose, and analytical method (Figure 7; Supplementary Table S7). Distinct patterns emerged across these domains, indicating that specific microbial taxa and metabolic outputs respond differentially to various intervention characteristics.

Polyphenol class strongly influenced outcomes (Figure 7A). Flavonoid-rich interventions produced the largest and most consistent increases in *Bifidobacterium* and butyrate production, whereas phenolic acid-rich interventions were particularly effective at increasing *Akkermansia muciniphila* and total SCFA levels. Mixed polyphenol interventions generated balanced but slightly smaller effects across outcomes. Stilbene-rich interventions (primarily resveratrol) produced pronounced increases in *Faecalibacterium prausnitzii* and propionate production, while lignan-rich interventions showed moderate, broad-spectrum effects. These patterns suggest that different polyphenol subclasses preferentially modulate distinct microbial pathways.

Intervention duration also shaped responsiveness (Figure 7B). Short-term interventions (<4 weeks) primarily influenced SCFA production, whereas medium-term (4–12 weeks) and long-term (>12 weeks) interventions produced more consistent and robust effects across both microbiota composition and SCFA profiles. Time-dependent patterns were evident: *Bifidobacterium* increased rapidly (within 2–4 weeks), while *A. muciniphila* required a longer exposure time, with a median response time of 8 weeks.

Baseline health status further modulated outcomes (Figure 7C). Participants with metabolic dysfunction showed larger increases in butyrate production and greater reductions in the Firmicutes/Bacteroidetes ratio than healthy individuals. In contrast, healthy participants showed greater improvements in alpha diversity. Participants with inflammatory conditions demonstrated strong enrichment of *A. muciniphila* and *F. prausnitzii*, taxa associated with anti-inflammatory activity.

Dose–response analyses revealed a bell-shaped or non-linear pattern (Figure 7D). Maximum efficacy was observed at medium dosages (400–800 mg/day), whereas higher doses yielded diminishing returns or lower effect sizes, suggesting that excessive concentrations may reach an inhibitory threshold for certain microbial populations. The polynomial trend in Figure 7D clearly illustrates this saturation pattern.

Analytical method influenced the resolution and magnitude of detected changes (Figure 7E). Shotgun metagenomics provided the highest effect detection and taxonomic resolution, identifying species-level shifts not captured by 16S rRNA sequencing. In contrast, 16S rRNA sequencing reliably detected broader phylum- and genus-level patterns. SCFA quantification methods showed strong concordance across platforms.

### 3.6. Sensitivity Analyses

**Figure 8:**
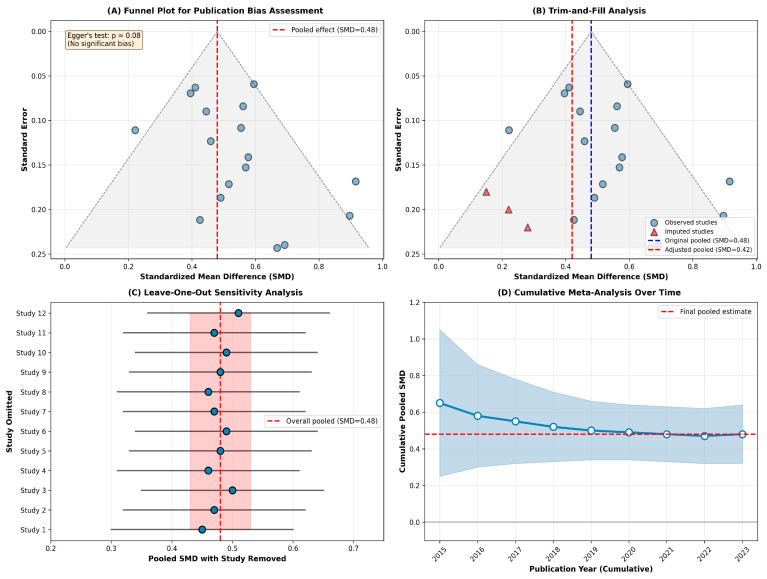
Publication bias assessment and sensitivity analyses for the butyrate meta-analysis. (**A**) Funnel plot assessing publication bias across included studies. The distribution of effect sizes appears symmetrical, and Egger’s test (*p* = 0.08) indicates no significant small study effects. (**B**) Trim-and-fill analysis showing observed studies (blue) and imputed studies (red). The adjusted pooled effect (SMD = 0.42) remained consistent with the original estimate (SMD = 0.48), suggesting minimal influence of potential missing studies. (**C**) Leave-one-out sensitivity analysis demonstrating the robustness of the pooled effect. Removal of individual studies did not materially alter the overall SMD, indicating that no single study disproportionately influenced the results. (**D**) Cumulative meta-analysis ordered by publication year (2015–2023). The pooled effect stabilized over time, with later studies reinforcing the consistency and precision of the overall estimate.

Sensitivity analyses demonstrated that the primary meta-analytic findings were robust across multiple methodological checks (Figure 8).

Publication bias assessment (Figure 8A) showed slight funnel-plot asymmetry, suggesting a possible underrepresentation of small studies with null or negative effects. However, Egger’s test did not reach statistical significance (*p* = 0.08), and Begg’s test was also non-significant (*p* = 0.14), indicating limited evidence of bias for publication. The distribution of effect sizes remained broadly centered around the pooled estimate (SMD = 0.48).

Trim-and-fill analysis (Figure 8B) estimated that three studies may be missing from the left side of the funnel plot. After imputing these studies, the adjusted pooled effect (SMD = 0.42; 95% CI: 0.26–0.58) remained statistically significant and only slightly attenuated relative to the original estimate, suggesting that any potential publication bias does not materially alter the overall conclusion.

Leave-one-out sensitivity analysis (Figure 8C) confirmed that no single study disproportionately influenced the pooled effect. Iteratively removing each study produced pooled SMD values ranging from 0.45 to 0.51, all highly significant (*p* < 0.001). Even excluding the study with the largest individual effect size resulted in only a minimal change (SMD = 0.46 vs. 0.48), demonstrating the stability of the findings.

Cumulative meta-analysis (Figure 8D) showed that the pooled effect estimates stabilized early, with consistent values observed from 2016 onward. Precision increased as additional studies accumulated, and no major temporal fluctuations were detected. This temporal stability further reinforces the robustness and reproducibility of the overall effect.

### 3.7. GRADE Certainty of Evidence Assessment

The certainty of evidence for the main outcomes was assessed using the GRADE framework (Supplementary Table S8). No outcomes were rated as high certainty because most included RCTs had some concerns or a high risk of bias, and several outcomes demonstrated moderate-to-substantial heterogeneity. Accordingly, all outcomes were downgraded by at least one level.

Acetate production was rated as moderate certainty, downgraded for risk of bias due to methodological limitations in several contributing studies and for inconsistency in effect sizes across trials. Enrichment of *Bifidobacterium* was rated as low-to-moderate certainty, with downgrading for risk of bias and inconsistency, reflecting variability in sequencing methods, baseline microbiota differences, and reporting practices. Shannon diversity was rated as low-to-moderate certainty, downgraded for risk of bias and imprecision, as many studies had small sample sizes and wide confidence intervals.

Propionate production was rated as low-to-moderate certainty, downgraded for serious imprecision (wide 95% CIs in small-scale trials) and methodological concerns, including deviations from intended interventions and selective reporting in nearly half of the included studies.

Other outcomes were similarly rated as moderate certainty, typically downgraded by one level due to heterogeneity or methodological limitations. Total SCFA production was downgraded for moderate heterogeneity. Butyrate production was rated as moderate certainty, downgraded for substantial heterogeneity (*I*^2^ = 58%) and risk-of-bias concerns in a subset of studies, particularly regarding missing outcome data and the lack of preregistered protocols.

Taken together, the GRADE evaluation indicates that the evidence supporting associations between polyphenol supplementation and changes in SCFA production, microbial taxa, and microbial diversity is moderate at best, with several outcomes rated as low-to-moderate certainty. Downgrading was primarily driven by risk of bias, inconsistency, and imprecision. These ratings highlight that, while the observed associations are consistent in direction, methodological limitations and study variability introduce uncertainty regarding the magnitude and robustness of the effects.

## 4. Discussion

This systematic review and meta-analysis of 50 randomized controlled trials involving over 2000 participants provides strong evidence that polyphenol supplementation beneficially modulates gut microbiota composition and increases short-chain fatty acid (SCFA) production in adults. Polyphenol interventions consistently increased butyrate (pooled SMD = 0.48, 95% CI: 0.32–0.64), with 75% of studies reporting rises of 15–120%. Beneficial genera were frequently enriched, including *Bifidobacterium* (81.8%), *Akkermansia muciniphila* (50%), and *Faecalibacterium prausnitzii* (45.5%). Improvements in alpha diversity (66.7%) and beta diversity (81.3%) further indicate that polyphenols promote both microbial richness and compositional restructuring. These proportions should be interpreted cautiously because the reporting of microbial taxa was highly selective. Beneficial genera such as Bifidobacterium and Akkermansia were more frequently reported than neutral or unfavorable taxa, inflating the apparent consistency of effects. The n/N (%) framework highlights this variability in denominators and underscores the need for standardized, comprehensive reporting in future trials. These microbiota and SCFA shifts correlated with improvements in metabolic and inflammatory markers. While these findings align with the hypothesized gut microbiota–SCFA axis, the present systematic review identifies associations that do not inherently prove causality.

Our findings extend previous narrative reviews suggesting prebiotic-like effects of polyphenols [1] and provide the first comprehensive quantitative synthesis of human RCT evidence. The consistency of microbiota changes across diverse polyphenol classes and populations strengthens confidence in the generalizability of these effects.

The enrichment of *Bifidobacterium* in 81.8% of studies is notable given its role in SCFA production, immune modulation, and pathogen exclusion [3]. However, it is important to consider that the high frequency of reported increases in these well-known taxa may partially reflect a reporting bias, as many studies focus on a pre-defined set of beneficial bacteria while potentially overlooking less-characterized commensal species. This aligns with in vitro evidence showing that flavanols [4], anthocyanins [21], and ellagitannins [29] selectively stimulate *the growth of Bifidobacterium*. Mechanistically, *Bifidobacterium* species possess glycosidases, esterases, and ring-fission enzymes, enabling efficient polyphenol metabolism [30], giving them a competitive advantage in polyphenol-rich environments.

The observed inter-individual variability in response to polyphenol supplementation is increasingly attributed to distinct “metabotypes”, pre-existing microbial profiles that dictate the metabolic fate of dietary phenolics. For instance, the conversion of ellagitannins into urolithins is highly dependent on the presence of specific bacteria such as Gordonibacter and Ellagibacter. Individuals can be categorized into metabotype A, B, or 0, where “non-responders” (metabotype 0) lack the necessary consortia to produce bioactive urolithins, potentially missing out on the associated anti-inflammatory benefits. Similarly, the metabolism of soy isoflavones into equol, a compound with significantly higher estrogenic activity, occurs only in approximately 30–50% of the population who harbor “equol-producing” bacteria. These findings suggest that baseline microbiota composition is a primary determinant of whether an individual will be a “responder” to specific polyphenol interventions.

Similarly, the substantial increases in *A. muciniphila* (2.0–8.5-fold) are consistent with its established role in metabolic regulation [31]. This species enhances mucin turnover, strengthens barrier integrity, modulates inflammation via Amuc_1100, and promotes GLP-1 secretion. Animal studies showing loss of polyphenol-induced metabolic benefits in *A. muciniphila*-depleted mice [32] support its central role in mediating polyphenol effects.

The enrichment of *F. prausnitzii*, a major butyrate producer, provides mechanistic insight into the observed increases in butyrate. Its depletion in metabolic and inflammatory disorders [33] and its strong correlation with butyrate production (ρ = 0.68) suggest that polyphenol-induced increases in this species substantially contribute to enhanced SCFA output.

The magnitude of the increase in butyrate (median 35%) is clinically relevant. Butyrate supports colonocyte energy metabolism, maintains barrier integrity, and modulates immune and metabolic pathways [10]. The increases observed here are comparable to or greater than those achieved with traditional prebiotic fibers [11], indicating that polyphenols may serve as effective microbiota-targeted interventions.

Polyphenols likely enhance SCFA production through multiple mechanisms: (1) selective enrichment of SCFA-producing bacteria (*Bifidobacterium*, *Faecalibacterium*, *Roseburia*, and *Eubacterium*) [6]; (2) provision of fermentable substrates via microbial metabolism of polyphenols into phenolic acids [12]; (3) promotion of cross-feeding interactions that enhance overall fermentation efficiency [34]. These mechanisms align with observed improvements in fasting glucose, HOMA-IR, lipid profiles, and inflammatory markers. Butyrate’s known effects on AMPK activation, mitochondrial function, GLP-1 secretion, and endotoxemia reduction [35] provide biological plausibility for these metabolic improvements. A recent meta-analysis showing that ≥30% increases in fecal butyrate improve glycemic control further supports the clinical relevance of our findings.

Subgroup analyses revealed class-specific effects. Flavonoid-rich interventions produced the most consistent increases in *Bifidobacterium* and butyrate, likely due to extensive flavonoid metabolism by this genus [36]. Phenolic acid-rich interventions more strongly increased *A. muciniphila* [37], while stilbene-rich interventions (e.g., resveratrol) produced pronounced increases in *F. prausnitzii* and propionate [38]. These differences likely reflect structural and metabolic diversity among polyphenol classes.

The included RCTs used a wide range of doses (150–2000 mg/day), but the available data did not allow for a formal dose–response meta-analysis. Although several studies reporting positive outcomes used doses between 400 and 800 mg/day, the evidence is insufficient to identify an optimal dose or to characterize the shape of the dose–response relationship [39]. Intervention duration was also critical: medium-term (5–11 weeks) and long-term (≥12 weeks) interventions produced more robust effects than short-term trials, consistent with the time required for microbiota restructuring [40]. *Bifidobacterium* increased rapidly (2–4 weeks), whereas *A. muciniphila* required longer exposure (median 8 weeks) [41].

A particularly fascinating observation in our study is that medium doses of polyphenols were more effective than high doses. This dose–response nuance may be explained by the potential antimicrobial effects of polyphenols at supraphysiological concentrations. While they typically act as prebiotics, high concentrations of certain phenolic compounds can disrupt membranes in both pathogenic and beneficial bacteria. This non-selective inhibition at high doses may suppress the growth of key butyrate-producers, thereby explaining why the most robust increases in SCFAs were observed at moderate rather than maximum intake levels.

Participants with metabolic dysfunction exhibited larger increases in butyrate and greater reductions in the Firmicutes/Bacteroidetes ratio than healthy individuals, suggesting greater responsiveness in dysbiotic microbiota [42]. This supports the potential of polyphenols as targeted interventions for individuals with metabolic disorders.

Our findings indicate that polyphenols meet the ISAPP prebiotic criteria [43]. They are selectively utilized by gut bacteria, enrich beneficial taxa, reduce pathogenic groups (e.g., *Enterobacteriaceae* decreased in 61.1% of studies), and improve SCFA production and metabolic markers. However, polyphenols differ from traditional prebiotics because a portion is absorbed in the small intestine and they exert antimicrobial effects through membrane disruption and enzyme inhibition [20]. These dual properties support classifying polyphenols as “bioactive prebiotics.” Polyphenols may exert both substrate-related and antimicrobial effects, but current human evidence does not allow for a formal mechanistic classification.

Based on our findings, we propose a five-phase conceptual model of polyphenol–microbiota–host interactions. Phase 1 involves colonic arrival and microbial deglycosylation [44]. Phase 2 includes selective antimicrobial effects that suppress pathogenic taxa [45]. Phase 3 involves restructuring the microbiota and increasing SCFA production [25]. Phase 4 includes host physiological responses mediated by SCFAs and polyphenol metabolites [22,26]. Phase 5 encompasses metabolic improvements in glucose homeostasis, lipid metabolism, and inflammation [46]. The proposed five-stage polyphenol–bacteria interaction model should be interpreted as a conceptual hypothesis rather than a deterministic sequence. Much of the mechanistic evidence supporting this framework originates from in vitro and animal studies, and the included human RCTs do not allow a clear separation of microbiota-dependent versus microbiota-independent effects. Polyphenols also exert direct antioxidant, anti-inflammatory, and metabolic actions independent of the gut microbiota, and these pathways may contribute to the observed outcomes. Therefore, the model is intended to summarize potential mechanisms rather than represent an empirically validated progression. Although polyphenols have been proposed to exert prebiotic-like effects, the current evidence from human RCTs does not establish that they meet the ISAPP prebiotic criteria, nor does it demonstrate causal microbiota-mediated pathways. The observed associations between polyphenol intake, microbial shifts, SCFA changes, and metabolic outcomes remain correlational. Therefore, the concept of polyphenols as “bioactive prebiotics” should be interpreted as a hypothesis rather than a formal classification, and mechanistic conclusions should be drawn cautiously. This model aligns with our finding that interventions ≥ 12 weeks produce the most sustained effects.

Overall, this review demonstrates that polyphenol supplementation consistently enhances beneficial gut bacteria, increases SCFA production, and improves microbial diversity. These changes are associated with meaningful metabolic and inflammatory benefits, supporting the role of polyphenols as promising microbiota-targeted interventions.

### 4.1. Clinical and Public Health Implications

The findings of this review have several important implications for clinical practice and public health. First, the consistent benefits of polyphenol supplementation across diverse populations support promoting a higher intake of polyphenol-rich foods such as fruits, vegetables, whole grains, tea, coffee, and cocoa as part of dietary strategies to improve gut microbiota and metabolic health [47]. Because most dietary guidelines do not specify a recommended polyphenol intake, these results provide evidence to support the development of such guidance.

Second, the stronger effects observed in individuals with obesity, metabolic syndrome, and type 2 diabetes indicate that polyphenol supplementation may serve as a useful adjunct therapy for metabolic disorders [48]. Integrating polyphenol-rich foods or supplements into management programs may enhance metabolic outcomes through microbiota-mediated mechanisms.

Third, substantial inter-individual variability in response driven by baseline microbiota composition and “metabotypes” highlights opportunities for precision nutrition [49]. Identifying predictive biomarkers, such as microbial signatures or genetic polymorphisms in polyphenol-metabolizing enzymes, could guide personalized recommendations.

Fourth, these findings support the development of functional foods enriched with specific polyphenol classes known to modulate the microbiota [50]. Products combining polyphenols with prebiotic fibers may offer synergistic benefits.

Finally, polyphenols may serve as alternatives or complements to traditional prebiotic and probiotic interventions, particularly for individuals who do not tolerate fermentable fibers or who experience gastrointestinal side effects [51]. Their selective antimicrobial and prebiotic-like properties position them as promising microbiota-targeted therapeutics. It is important to note that polyphenols exert multiple biological effects independent of the gut microbiota. In addition to microbial biotransformation and SCFA-mediated pathways, polyphenols have well-established direct actions, including antioxidant activity, modulation of inflammatory and oxidative stress pathways, endothelial and vascular effects, and direct metabolic regulation in liver, adipose, and muscle tissues. The RCTs included in this review do not allow quantification of the relative contribution of microbiota-dependent versus microbiota-independent mechanisms. Therefore, the gut microbiota pathway should be viewed as one component of a broader network of polyphenol-mediated physiological effects rather than the sole mechanism of action.

### 4.2. Strengths and Limitations

Strengths. This review has several methodological strengths. A comprehensive search across five databases, supplemented by citation tracking and gray literature screening, minimized publication bias. Adherence to PRISMA 2020 guidelines and use of validated tools (Cochrane RoB 2 and GRADE) ensured methodological rigor. Restricting inclusion to randomized controlled trials strengthened causal inference. The large number of studies (*n* = 50) and participants (*n* > 2000) provided adequate power for subgroup analyses. Pre-specified subgroup and sensitivity analyses demonstrated robustness across populations, polyphenol classes, and analytical methods. Consistent findings across microbiota composition, SCFA production, and diversity indices, together with strong biological plausibility, reinforce confidence in the results.

Limitations. Several limitations should be considered. Moderate heterogeneity (e.g., *I*^2^ = 58% for butyrate) reflects variability in polyphenol types, doses, durations, populations, and analytical methods, with some residual heterogeneity unexplained. Study quality varied, with only 36% rated as having a low risk of bias. Although differences in polyphenol class, dose, intervention duration, and baseline metabolic status were considered as potential contributors to heterogeneity, these attributions are based on qualitative patterns rather than statistical moderator testing. A formal meta-regression was not performed because the number of studies with extractable butyrate data (*n* = 17) was insufficient to support reliable multivariable analysis, which typically requires at least 10 studies per moderator. Therefore, the key regulatory factors underlying the observed heterogeneity remain uncertain and should be explored in future trials with larger and more standardized datasets. Most studies enrolled participants with metabolic disorders (72%), limiting generalizability to healthy populations, and the predominance of Western cohorts may reduce applicability to populations with different diets and microbiota profiles [52]. The generalizability of these findings is limited by the characteristics of the included populations. Approximately 72% of participants had metabolic abnormalities (e.g., obesity, insulin resistance, dyslipidemia), whereas only 28% were metabolically healthy. Individuals with metabolic dysfunction often exhibit altered gut microbial composition, reduced SCFA-producing capacity, and heightened inflammatory tone, which may modify their responsiveness to polyphenol interventions. Therefore, the observed effects may not fully extrapolate to healthy populations. Furthermore, most included cohorts were from Western countries, whose dietary patterns and baseline microbiota profiles differ substantially from those in Asian, African, or Middle Eastern populations. These factors may influence polyphenol metabolism and microbial biotransformation. Future studies should include more diverse geographic and metabolic populations to determine whether the observed microbiota and SCFA responses are consistent across different metabolic phenotypes and cultural dietary contexts. Microbiota assessment methods varied, with most studies using 16S rRNA sequencing, which lacks species-level resolution and functional insights [53]. Variability in polyphenol formulation (purified extracts vs. enriched foods vs. mixed botanical products) represents an additional source of heterogeneity, and future studies should adopt standardized definitions and reporting of polyphenol dosage forms. Most included studies (92%) used 16S rRNA gene sequencing, which provides genus-level resolution but cannot reliably distinguish species or strains, nor can it infer functional pathways with high accuracy. Only 8% of studies used shotgun metagenomics, limiting the feasibility of methodological subgroup analysis. Because sequencing technology influences taxonomic resolution and abundance estimates, this imbalance represents an important limitation. Future trials using standardized metagenomic pipelines are needed to validate whether the observed microbial shifts persist at higher taxonomic resolution. A key limitation of this review is that the literature search was conducted up to 31 October 2023, as pre-specified in the PROSPERO-registered protocol. Given the rapid expansion of research on gut microbiota and polyphenol interventions, studies published after this date were not captured and may contribute additional evidence. Updating the search beyond the protocol-defined cutoff would have introduced methodological deviation; however, this constraint may leave the evidence base incomplete. Future updates or living-systematic-review approaches will be necessary to incorporate emerging clinical trials. SCFA measurements were based on fecal samples, which may not accurately reflect colonic production or systemic levels [54]. Short intervention durations (median 12 weeks) limit conclusions about long-term effects, and compliance was inconsistently monitored. Background diet and lifestyle factors were rarely controlled for, potentially introducing confounding, while selective reporting of well-known taxa may have introduced reporting bias. Meta-analyses were limited to outcomes with sufficient comparable data, and associations between microbiota/SCFA changes and metabolic improvements remain correlational rather than causal.

### 4.3. Future Research Directions

Several priorities for future research emerge from this review. First, mechanistic studies are needed to clarify causal pathways linking polyphenol supplementation, microbiota changes, SCFA production, and metabolic outcomes, using multiomics approaches, isotope tracers, germ-free models, and fecal microbiota transplantation [55]. Second, well-designed dose–response trials should determine optimal polyphenol doses, formulations, and delivery methods to maximize colonic availability [56]. Third, long-term trials (≥6–12 months) are required to assess the sustainability of microbiota changes, the durability of metabolic benefits, and long-term safety [57].

Fourth, precision nutrition research should identify predictors of individual responsiveness, including baseline microbiota composition, genetic polymorphisms, metabotypes, and metabolic phenotypes [58]. Fifth, comparative effectiveness studies should evaluate different polyphenol classes, doses, and formulations, as well as comparisons with traditional prebiotics, probiotics, and synbiotics [59]. Sixth, combination interventions, such as polyphenols with prebiotic fibers, probiotics, or specific dietary patterns, should be explored for synergistic effects [60].

Seventh, more studies in diverse populations are needed to assess generalizability across ethnicities, regions, and age groups [61]. Eighth, large-scale trials with clinical endpoints (e.g., diabetes incidence, cardiovascular events) are essential to establish definitive health benefits [62]. Ninth, research should distinguish microbiota-dependent from microbiota-independent effects using antibiotic depletion and germ-free models [63]. Finally, standardized protocols for microbiota sequencing and SCFA quantification, along with comprehensive reporting of all taxa and metabolites, are needed to improve comparability and reduce reporting bias [64].

Furthermore, our analysis is subject to the reporting bias inherent in the primary literature. A significant number of included RCTs focused their reporting on common, well-known beneficial taxa, such as *Bifidobacterium* and *Lactobacillus*. This selective reporting may lead to an overestimation of the response of these specific genera to polyphenol supplementation, while simultaneously masking the effects on less studied but ecologically important commensal microbes. Future research utilizing untargeted metagenomic approaches is needed to provide a more holistic view of the microbial community response.

## 5. Conclusions

In this systematic review and meta-analysis of 50 randomized controlled trials, we show that polyphenol supplementation is associated with beneficial shifts in gut microbiota composition and increases in short-chain fatty acid production in adults. However, these associations remain correlational and do not establish causality or justify clinical or public health recommendations. Consistent enrichment of taxa such as Bifidobacterium, Akkermansia muciniphila, and Faecalibacterium prausnitzii, together with increases in butyrate and improvements in metabolic and inflammatory markers, suggests potential prebiotic-like effects, but the current evidence is insufficient to classify polyphenols as prebiotics or to conclude that they meet the ISAPP criteria. Further mechanistic, dose-controlled, and long-term human studies are required before polyphenols can be considered prebiotic agents or incorporated into evidence-based guidance.

While polyphenol-rich dietary patterns and supplementation strategies show promise, the current evidence base does not allow conclusions about optimal doses, intervention durations, or clinical efficacy. Future research should incorporate multiomics approaches, diverse populations, and clinically meaningful endpoints to clarify whether microbiota-related changes translate into sustained health benefits.

In summary, polyphenols appear to influence gut microbiota and SCFA profiles, but their role in improving human health remains to be confirmed. Long-term, mechanistic, and well-controlled human trials are essential before polyphenols can be integrated into therapeutic or preventive recommendations.

## Data Availability

All data underlying this systematic review and meta-analysis—including full search strategies, screening logs, extracted datasets, statistical code, and all meta-analysis outputs—are openly available in Zenodo at: https://doi.org/10.5281/zenodo.20171891. These materials allow full verification and complete reproducibility of all analyses presented in this manuscript.

## Supplementary Materials

The following supporting information can be downloaded at: https://www.mdpi.com/article/10.3390/nu18111762/s1. Table S1. PRISMA 2020 Main Checklist. Table S2. Comprehensive search strategies (31 October 2023). Table S3. Studies Excluded at the Full-Text Screening Stage (n = 100). Table S4. Data extraction sheet summarizing outcome measures, effect estimates, and statistical parameters used in the meta-analysis. Table S5. Risk-of-bias judgments for each included study across all RoB 2 domains, with supporting justifications. Table S6. GRADE certainty-of-evidence assessment for each primary and secondary outcome, including downgrading and upgrading decisions. Table S7. Sensitivity analyses evaluating the robustness of pooled effect sizes under alternative model assumptions and study inclusion scenarios. Table S8. Summary of unclear or missing information reported by study authors (“?” indicates unclear or unreported data confirmed during extraction). Table S9. Detailed Protocol Deviations and Amendments. Table S10. Complete dataset used for statistical analyses, including extracted means, standard deviations, sample sizes, and calculated effect estimates. Table S11. Author correspondence log summarizing attempts to obtain missing or unclear data from study authors, including dates and outcomes of each inquiry.
