# Supplementary material for: Effects of Polyphenol Supplementation on Gut Microbiota Composition and Fecal Short-Chain Fatty Acids: A Systematic Review and Meta-Analysis of Randomized Controlled Trials
Source: Nutrients. 2026 May 30;18(11):1762. doi: 10.3390/nu18111762 (PMC13258798; doi:10.3390/nu18111762)
Supplement: Supplementary file 1 [file nutrients-18-01762-s001.zip › Supplementary Table S2.pdf]

SUPPLEMENTARY TABLE S2: COMPREHENSIVE SEARCH STRATEGIES (OCTOBER 31, 2023)

Database	Search Query Strategy	Results (Initial)
PubMed / MEDLINE	("polyphenols"[MeSH] OR "flavonoids"[MeSH] OR "phenolic acids"[MeSH] OR "proanthocyanidins"[MeSH] OR "resveratrol"[MeSH] OR "catechin"[MeSH] OR "quercetin"[MeSH] OR "stilbenes"[MeSH]) AND ("microbiota"[MeSH] OR "gastrointestinal microbiome"[MeSH] OR "dysbiosis"[MeSH] OR "short-chain fatty acids"[MeSH] OR "butyric acid"[MeSH] OR "propionic acid"[MeSH] OR "acetic acid"[MeSH]) AND ("randomized controlled trial"[Publication Type] OR "randomized"[Title/Abstract] OR "placebo"[Title/Abstract])	432
Scopus	TITLE-ABS-KEY((polyphenol* OR flavonoid* OR "phenolic acid*" OR anthocyanin* OR resveratrol OR quercetin OR catechin OR curcumin OR stilbene*) AND (microbiota OR microbiome OR "gut flora" OR "short-chain fatty acid*" OR SCFA* OR butyrate OR propionate OR acetate) AND ("randomized controlled trial" OR RCT OR "placebo-controlled" OR trial))	518
Web of Science	TS=((polyphenol* OR flavonoid* OR "phenolic acid*" OR anthocyanin* OR resveratrol OR quercetin OR catechin OR curcumin OR stilbene*) AND (microbiota OR microbiome OR "gut flora" OR "short-chain fatty acid*" OR SCFA* OR butyrate OR propionate OR acetate) AND ("randomized controlled trial" OR RCT OR "placebo-controlled" OR trial))	385
Embase	('polyphenol'/exp OR 'flavonoid'/exp OR 'phenolic acid'/exp OR 'anthocyanin'/exp OR 'resveratrol'/exp OR 'quercetin'/exp OR 'catechin'/exp OR 'curcumin'/exp OR 'stilbene'/exp) AND ('microbiota'/exp OR 'intestine flora'/exp OR 'short chain fatty acid'/exp OR 'butyrate'/exp OR 'propionate'/exp OR 'acetate'/exp) AND ('randomized controlled trial'/exp OR 'randomization'/exp OR 'double blind procedure'/exp)	267
Cochrane CENTRAL	([mh "polyphenols"] OR [mh "flavonoids"] OR [mh "phenolic acids"] OR [mh "proanthocyanidins"] OR [mh "resveratrol"] OR [mh "catechin"] OR [mh "quercetin"] OR [mh "stilbenes"]) AND ([mh "microbiota"] OR [mh "gastrointestinal microbiome"] OR [mh "short-chain fatty acids"] OR [mh "butyric acid"] OR [mh "propionic acid"] OR [mh "acetic acid"])	124
Grey Lit / Manual	ProQuest, Google Scholar, trial registries, reference lists, and citation tracking	186
TOTAL UNIQUE	AFTER DE-DUPLICATION (Covidence)	1,433
