# Supplementary material for: Effects of Polyphenol Supplementation on Gut Microbiota Composition and Fecal Short-Chain Fatty Acids: A Systematic Review and Meta-Analysis of Randomized Controlled Trials
Source: Nutrients. 2026 May 30;18(11):1762. doi: 10.3390/nu18111762 (PMC13258798; doi:10.3390/nu18111762)
Supplement: Supplementary file 1 [file nutrients-18-01762-s001.zip › Supplementary Table S3.pdf]

Supplementary Table S3: Studies Excluded at the Full-Text Screening Stage (n=100)

Category of Exclusion	Count (n)	Primary Reason(s) for Exclusion
Wrong Intervention	35	Combination with prebiotics/probiotics (synbiotics); Not a polyphenol-focused intervention
Wrong Study Design	22	Non-randomized trials; Observational studies; Case reports; Narrative reviews
Wrong Population	18	Pediatric populations (<18 years); Pregnant/lactating women; Animal studies not excluded at abstract stage
Wrong Outcome	15	No reported data on gut microbiota composition or fecal SCFA concentrations
Short Duration	6	Intervention duration less than 2 weeks
Recent Antibiotics	4	Participants received antibiotics within 3 months of the study
TOTAL EXCLUDED	100	As documented in Figure 1 (PRISMA Flow Diagram)

Detailed List of Representative Excluded Studies

Study ID	Reason for Exclusion
Thompson et al. (2022)	Wrong Intervention: Polyphenols combined with inulin (synbiotic)
García-Martínez et al. (2021)	Wrong Study Design: Single-arm pilot study (non-randomized)
Smith & Jones (2019)	Wrong Outcome: Focused on plasma polyphenols only; no microbiota data
Miller et al. (2020)	Wrong Population: Subjects were children (aged 5-12)
White et al. (2018)	Short Duration: Intervention was a single dose (acute study)
