# Supplementary material for: Effects of Polyphenol Supplementation on Gut Microbiota Composition and Fecal Short-Chain Fatty Acids: A Systematic Review and Meta-Analysis of Randomized Controlled Trials
Source: Nutrients. 2026 May 30;18(11):1762. doi: 10.3390/nu18111762 (PMC13258798; doi:10.3390/nu18111762)
Supplement: Supplementary file 1 [file nutrients-18-01762-s001.zip › Supplementary Table S9.pdf]

Supplementary Table S9: Detailed Protocol Deviations and Amendments

This table documents all deviations from the original PROSPERO protocol (CRD42025642315) with their respective justifications, as required by PRISMA 2020 reporting standards.

Item	Original Protocol Specification	Actual Review Implementation	Scientific Justification
Search Date	Search planned until September 2023	Extended to October 31, 2023	To incorporate the most recent high-impact trials published during the final analysis phase.
Reviewer 2	Independent reviewer to be confirmed	SA (Primary) and MZ (Independent Verification)	Personnel finalized and trained before the screening stage commenced.
Meta-Analysis	Only if $I^2 < 75\%$	Random-effects meta-analysis conducted regardless of I^2	Used subgroup analysis and meta-regression to explore and explain observed heterogeneity.
Additional Outcomes	SCFA and Microbiota only	Inclusion of secondary metabolic markers (glucose, lipids, weight)	Added to enhance clinical relevance and provide a broader context of health implications.
Subgroup Analysis	Age and health status only	Added intervention duration and analytical method subgroups	These variables were identified as significant contributors to variance during data extraction.
Software	RevMan 5.4	R v4.2.0 and Python v3.10	Required for advanced statistical modeling, custom visualizations (300 DPI), and data processing.
Data Verification	10% re-extraction	20% re-extraction	Enhanced quality assurance for numerical and graphical data (WebPlotDigitizer v4.5).
GRADE Assessment	Planned for primary outcomes	Conducted for all primary and secondary outcomes	To provide comprehensive evidence certainty ratings for all clinical findings.
